# Supplementary material for: Mechanisms of NLRP3 activation and inhibition elucidated by functional analysis of disease-associated variants
Source: Nat Immunol. 2025 Feb 10;26(3):511–23. doi: 10.1038/s41590-025-02088-9 (PMC11876074; doi:10.1038/s41590-025-02088-9)
Supplement: Supplementary file 1 — Reporting Summary [file 41590_2025_2088_MOESM1_ESM.pdf]

nature portfolio

Corresponding author(s): Seth L. Masters

Last updated by author(s): 2025-Jan-04

Reporting Summary

Nature Portfolio wishes to improve the reproducibility of the work that we publish. This form provides structure for consistency and transparency in reporting. For further information on Nature Portfolio policies, see our Editorial Policies and the Editorial Policy Checklist.

Statistics

For all statistical analyses, confirm that the following items are present in the figure legend, table legend, main text, or Methods section.

n/a	Confirmed	
☐	☒	The exact sample size (*n*) for each experimental group/condition, given as a discrete number and unit of measurement
☐	☒	A statement on whether measurements were taken from distinct samples or whether the same sample was measured repeatedly
☐	☒	The statistical test(s) used AND whether they are one- or two-sided<br>*Only common tests should be described solely by name; describe more complex techniques in the Methods section.*
☒	☐	A description of all covariates tested
☒	☐	A description of any assumptions or corrections, such as tests of normality and adjustment for multiple comparisons
☐	☒	A full description of the statistical parameters including central tendency (e.g. means) or other basic estimates (e.g. regression coefficient) AND variation (e.g. standard deviation) or associated estimates of uncertainty (e.g. confidence intervals)
☐	☒	For null hypothesis testing, the test statistic (e.g. F, t, r) with confidence intervals, effect sizes, degrees of freedom and P value noted<br>*Give P values as exact values whenever suitable.*
☒	☐	For Bayesian analysis, information on the choice of priors and Markov chain Monte Carlo settings
☒	☐	For hierarchical and complex designs, identification of the appropriate level for tests and full reporting of outcomes
☐	☒	Estimates of effect sizes (e.g. Cohen's d, Pearson's r), indicating how they were calculated

Our web collection on statistics for biologists contains articles on many of the points above.

Software and code

Policy information about availability of computer code

Data collection	FACS: BD FACSDiva™ Software (v9.0); SpectroFlo (v3.2.1)<br>ELSA: MARS (V3.01 R2)<br>Imaging flow cytometry: IDEAS software (V6.2)
Data analysis	GraphPad Prism (version 9.5.1)<br>Flowjo (10.8.2)<br>R Studio (version 2024.04.2+764)<br>flowCore (release no. bioc_3.13, https://github.com/RGLab/flowCore)<br>flowAI (release no. flowise@1.4.0, https://github.com/FlowiseAI/Flowise)<br>openCyto (release no. bioc_3.13, https://github.com/RGLab/openCyto)<br>drda (Malyutina, A. et al 2023, https://github.com/albertopessia/drda)<br>data2bfactor (2003 Robert L. Campbell, https://peterslab.org/downloads.php)<br>Autospeck workflow (this study, https://github.com/Seth-Masters-Lab/Speck-Assay-Automated-Workflow.git)<br>ChimeraX (version 1.7)<br>Pymol (version 2.5.0, Schrödinger, LLC)<br>Dynamut (Rodrigues, C. H., et al. 2018)<br>DDmut (Zhou, Y., et al. 2023)<br>mCSM-lig (Pires, D. E., et al. 2016)<br>Jpred (v4, Cole, C., 2008)<br>ImageLab Software (v6.01)

Quanstudio Design and Analysis Software (v2.8)
ChatGPT (v4)

For manuscripts utilizing custom algorithms or software that are central to the research but not yet described in published literature, software must be made available to editors and reviewers. We strongly encourage code deposition in a community repository (e.g. GitHub). See the Nature Portfolio guidelines for submitting code & software for further information.

Data

Policy information about availability of data

All manuscripts must include a data availability statement. This statement should provide the following information, where applicable:

- Accession codes, unique identifiers, or web links for publicly available datasets
- A description of any restrictions on data availability
- For clinical datasets or third party data, please ensure that the statement adheres to our policy

Our data is deposited in publicly available data repositories including INFEVERS (identifier: NLRP3, https://infevers.umai-montpellier.fr) and GenIA (identifier: NLRP3, https://www.geniadb.net/app/ref/info.php?id=1380). Databases used in this study include: INFEVERS (identifier: NLRP3, https://infevers.umai-montpellier.fr/web/), ClinVar (identifier: NLRP3, https://www.ncbi.nlm.nih.gov/clinvar/), AlphaMissense (identifier: NLRP3, https://alphamissense.hegelab.org/search), gnomAD (v4.1.0, identifier: NLRP3, https://gnomad.broadinstitute.org/gene/ENSG00000162711?dataset=gnomad_r4). Datasets used in this study are available in the RCSB Protein Data Bank (https://www.rcsb.org) with accession-codes including PDB:8EJ4, 7PZC, 6NPY and 7PZD.

Research involving human participants, their data, or biological material

Policy information about studies with human participants or human data. See also policy information about sex, gender (identity/presentation), and sexual orientation and race, ethnicity and racism.

Reporting on sex and gender	Not applicable.
Reporting on race, ethnicity, or other socially relevant groupings	Not applicable.
Population characteristics	Not applicable.
Recruitment	Not applicable.
Ethics oversight	Human peripheral blood mononuclear cells (PBMCs) were sourced from the Victorian Blood Donor Registry under WEHI HREC approval 18/07.

Note that full information on the approval of the study protocol must also be provided in the manuscript.

Field-specific reporting

Please select the one below that is the best fit for your research. If you are not sure, read the appropriate sections before making your selection.

☒ Life sciences ☐ Behavioural & social sciences ☐ Ecological, evolutionary & environmental sciences

For a reference copy of the document with all sections, see nature.com/documents/nr-reporting-summary-flat.pdf

Life sciences study design

All studies must disclose on these points even when the disclosure is negative.

Sample size	Sample size, or the number of NLRP3 variants tested, id determined based on availability. NLRP3 variants tested in this study were sourced from publicly available datasets including INFEVERS and ClinVar. Additional variants were sourced from clinician in contact.
Data exclusions	FACS data were processed in a fully automated manner using the R code generated in this study. Sample with poor quality where the dose-response curve fitting failed to be generated by the code were excluded or labelled as out of scale where appropriate.
Replication	All experiments were independently repeated at least 2 times as indicated in figure legends. Where possible, findings were confirmed in different experimental systems.
Randomization	Transfected or reconstituted cells used in this study represent pre-defined experimental groups. These were created to test specific hypotheses regarding gene function and variant effects, making randomization unnecessary.
Blinding	Controls were used in each experimental conditions, and results were recorded automatically or analyzed through software and thus reducing the potential for subjective influence . Samples or cell lines were coded during experiments providing partial blinding measurement.

Reporting for specific materials, systems and methods

We require information from authors about some types of materials, experimental systems and methods used in many studies. Here, indicate whether each material, system or method listed is relevant to your study. If you are not sure if a list item applies to your research, read the appropriate section before selecting a response.

Materials & experimental systems

n/a	Involved in the study
☐	☒ Antibodies
☐	☒ Eukaryotic cell lines
☒	☐ Palaeontology and archaeology
☒	☐ Animals and other organisms
☒	☐ Clinical data
☒	☐ Dual use research of concern
☒	☐ Plants

Methods

n/a	Involved in the study
☒	☐ ChIP-seq
☐	☒ Flow cytometry
☒	☐ MRI-based neuroimaging

Antibodies

Antibodies used	NLRP3 (1:1000, rabbit, CST, 15101S)<br>NEK7 (1:1000,rabbit, CST, 3057S)<br>Tubulin (1:5000, rat, Santa Cruz Biotechnology, sc-53029)<br>β-actin conjugated with horseradish peroxidase (1:5000, Santa Cruz Biotechnology, SANTSC-47778HRP)<br>Goat anti-rat horseradish peroxidase-conjugated secondary antibodies (1:5000, Invitrogen, 31470)<br>Goat anti-Rabbit IgG (H+L) Secondary Antibody, HRP (1:5000, Invitrogen, 31460)
Validation	All antibodies were validated by the manufacturers and confirmed using knockout cells in this study (NLRP3, NEK7). Links for additional validation report:<br>NLRP3 (rabbit, CST, 15101S)<br>https://www.cellsignal.com/products/primary-antibodies/nlrp3-d4d8t-rabbit-mab/15101?srsltid=AfmBOorLKaVqqtz6o42ayCbJ8PT2ACME9jnPpFG38gvUeTdmer6w3Qtf<br>NEK7 (rabbit, CST, 3057S)<br>https://www.cellsignal.com/products/primary-antibodies/nek7-c34c3-rabbit-mab/3057?srsltid=AfmBOoq62uRPLP1WQ7P-3RA3Vl6qoZyc3_fTQMfZCrrmeD8mVh2C6Uik<br>Tubulin (rat, Santa Cruz Biotechnology, sc-53029)<br>https://www.scbt.com/p/alpha-tubulin-antibody-yl1-2?srsltid=AfmBOopMr1MgMVp83IFt3ucQ55hXaGc4c7mRLsdK8asbf5YBo69ybwxZ<br>β-actin-HRP (Santa Cruz Biotechnology, SANTSC-47778HRP)<br>https://www.scbt.com/p/beta-actin-antibody-c4?srsltid=AfmBOooldZZeATWlAMBYB5mVcMGlAikozpoIA7kcbJHIQ0y7k-NKM_v-<br>anti-rat horseradish peroxidase-conjugated secondary antibodies (Invitrogen, 31470)<br>https://www.thermofisher.com/antibody/product/Goat-anti-Rat-IgG-H-L-Secondary-Antibody-Polyclonal/31470<br>Goat anti-Rabbit IgG (H+L) Secondary Antibody, HRP (Invitrogen, 31460)<br>https://www.thermofisher.com/antibody/product/Goat-anti-Rabbit-IgG-H-L-Secondary-Antibody-Polyclonal/31460

Eukaryotic cell lines

Policy information about cell lines and Sex and Gender in Research

Cell line source(s)	Human embryonic kidney (HEK) 293T cells (ATCC CRL-3216)<br>THP-1 cells (ATCC TIB-202)<br>U937 (ATCC CRL-1593.2)
Authentication	All cell lines were authenticated using ATCC's human cell authentication service utilizing short tandem repeat (STR) profiling.
Mycoplasma contamination	All cell lines were confirmed negative for mycoplasma by PCR or MycoStrip (InvivoGen, rep-mysnc-100) throughout the duration of this study.
Commonly misidentified lines<br>(See ICLAC register)	No commonly misidentified cell lines were used.

Plants

Seed stocks	*Report on the source of all seed stocks or other plant material used. If applicable, state the seed stock centre and catalogue number. If plant specimens were collected from the field, describe the collection location, date and sampling procedures.*
Novel plant genotypes	*Describe the methods by which all novel plant genotypes were produced. This includes those generated by transgenic approaches, gene editing, chemical/radiation-based mutagenesis and hybridization. For transgenic lines, describe the transformation method, the number of independent lines analyzed and the generation upon which experiments were performed. For gene-edited lines, describe the editor used, the endogenous sequence targeted for editing, the targeting guide RNA sequence (if applicable) and how the editor was applied.*
Authentication	*Describe any authentication procedures for each seed stock used or novel genotype generated. Describe any experiments used to assess the effect of a mutation and, where applicable, how potential secondary effects (e.g. second site T-DNA insertions, mosiacism, off-target gene editing) were examined.*

Flow Cytometry

Plots

Confirm that:

☒ The axis labels state the marker and fluorochrome used (e.g. CD4-FITC).

☒ The axis scales are clearly visible. Include numbers along axes only for bottom left plot of group (a 'group' is an analysis of identical markers).

☒ All plots are contour plots with outliers or pseudocolor plots.

☒ A numerical value for number of cells or percentage (with statistics) is provided.

Methodology

Sample preparation	For flow cytometry, HEK293T cells stably expressing BFP-ASC were transfected with plasmids expressing GFP-NLRP3. Cells were collected and fixed in pre-chilled methanol or in pre-warmed 4% PFA and resuspended in PBS supplemented with 2% FCS and 5 mM EDTA. THP-1 cells were directly resuspended in PBS supplemented with 2% FCS and 5 mM EDTA.
Instrument	Amnis ImageStreamX MKII imaging flow cytometer<br>BD LSR Fortessa X20<br>Cytek Aurora
Software	IDEAS software (V6.2)<br>BD FACSDiva™ Software (v9.0)<br>SpectroFlo (v3.2.1)
Cell population abundance	At least 10,000 cells were acquired for each sample.
Gating strategy	Samples were gated for non-debri single cells based on FSC/SSC and then gated on ASC speck based on BFP-A/BFP-W and GFP with GFP-A/FSC-A.

☒ Tick this box to confirm that a figure exemplifying the gating strategy is provided in the Supplementary Information.
