# Supplementary figures and images for: Mechanisms of NLRP3 activation and inhibition elucidated by functional analysis of disease-associated variants
Source: Nat Immunol. 2025 Feb 10;26(3):511–23. doi: 10.1038/s41590-025-02088-9 (PMC11876074; doi:10.1038/s41590-025-02088-9)

Extended Data Fig. 3a

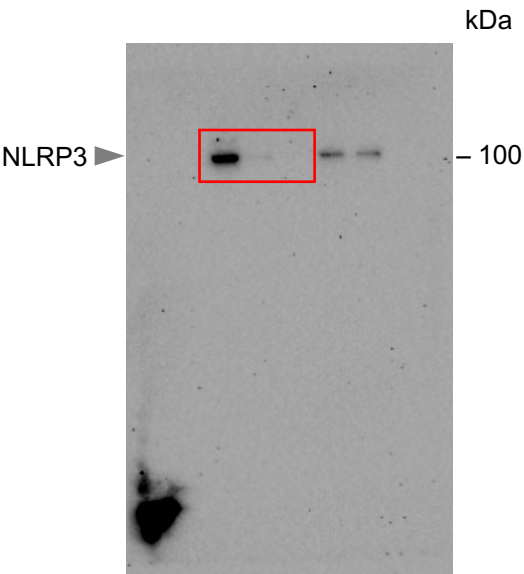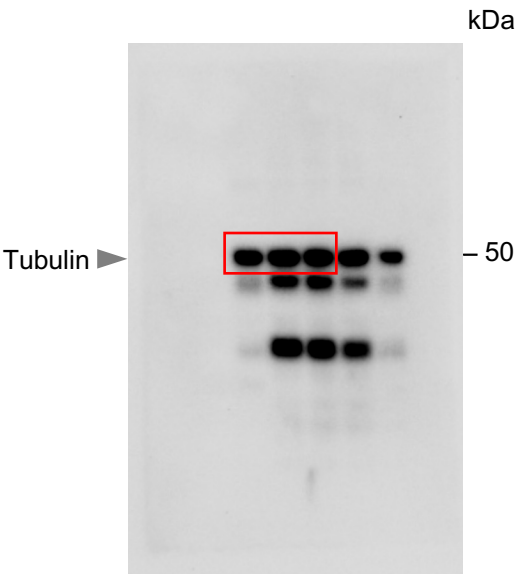

Supplement: Supplementary file 4 — Unprocessed immunoblots. [file 41590_2025_2088_MOESM4_ESM.pdf]

Extended Data Fig. 7a

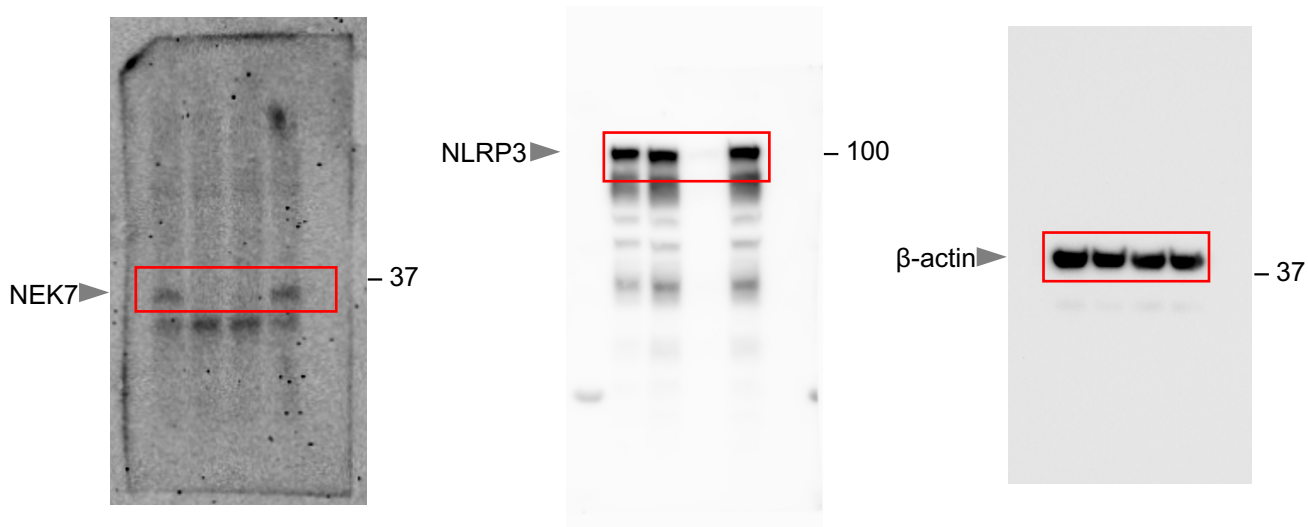

Supplement: Supplementary file 5 — Unprocessed immunoblots. [file 41590_2025_2088_MOESM5_ESM.pdf]
